# Supplementary material for: Fabrication and Performance Evaluation of Gelatin/Sodium Alginate Hydrogel-Based Macrophage and MSC Cell-Encapsulated Paracrine System with Potential Application in Wound Healing
Source: Int J Mol Sci. 2023 Jan 8;24(2):1240. doi: 10.3390/ijms24021240 (PMC9867201; doi:10.3390/ijms24021240)
Supplement: Supplementary file 1 [file ijms-24-01240-s001.zip › ijms-2154629-supplementary.pdf]

Fabrication and Performance Evaluation of Gelatin/Sodium Alginate Hydrogel-Based Macrophage and MSC Cell-Encapsulated Paracrine System with Potential Application in Wound Healing

[#]Hang Yao[1], [#]Xiaohui Yuan[1], Zhonglian Wu[1], Sumin Park[2], Wang Zhang[1, 2, 3], Hui Chong[1, *], Liwei Lin[2, *], Yuanzhe Piao[2, 3]

[1] School of Chemistry and Chemical Engineering, Yangzhou University, Yangzhou 225009, China

[2] Department of Applied Bioengineering, Graduate School of Convergence Science and Technology, Seoul Na-tional University, Seoul 08826, Republic of Korea

[3] Advanced Institutes of Convergence Technology, Suwon, Gyeonggi-do, 16229, Republic of Korea

[#] These authors contributed equally to this work.

[*]Correspondence:

lin-official@snu.ac.kr (L. L.); chonghui@yzu.edu.cn (H. C.)

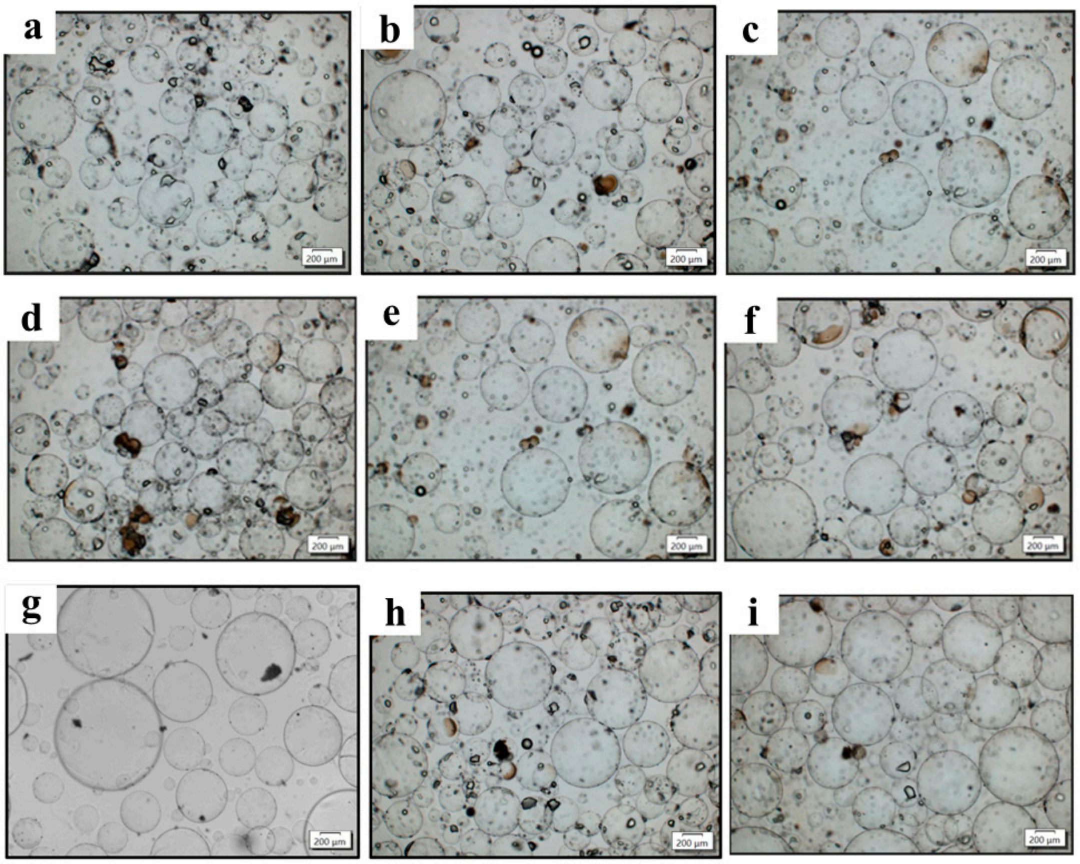

Figure S1. Photos of microsphere prepared under different conditions. a: reagent concentration of 1% (w/v), water-oil ratio of 1:2.5, stirring rate of 500-600 rpm. b: reagent concentration of 1.5% (w/v), water-oil ratio of 1:2.5, stirring rate of 500-600 rpm. c: reagent concentration of 2% (w/v), water-oil ratio of 1:2.5, stirring rate of 500-600 rpm. d: water-oil ratio of 1:2, reagent concentration of 1.5% (w/v), stirring rate of

500-600 rpm. e: water-oil ratio of 1:2.5, reagent concentration of 1.5% (w/v), stirring rate of 500-600 rpm. f: water-oil ratio of 1:3, reagent concentration of 1.5% (w/v), stirring rate of 500-600 rpm. g: stirring rate of 450-550 rpm, water-oil ratio of 1:2.5, reagent concentration of 1.5% (w/v). h: stirring rate of 500-600 rpm, water-oil ratio of 1:2.5, reagent concentration of 1.5% (w/v). i: stirring rate of 550-650 rpm, water-oil ratio of 1:2.5, reagent concentration of 1.5% (w/v). Scale bar = 200 mm.

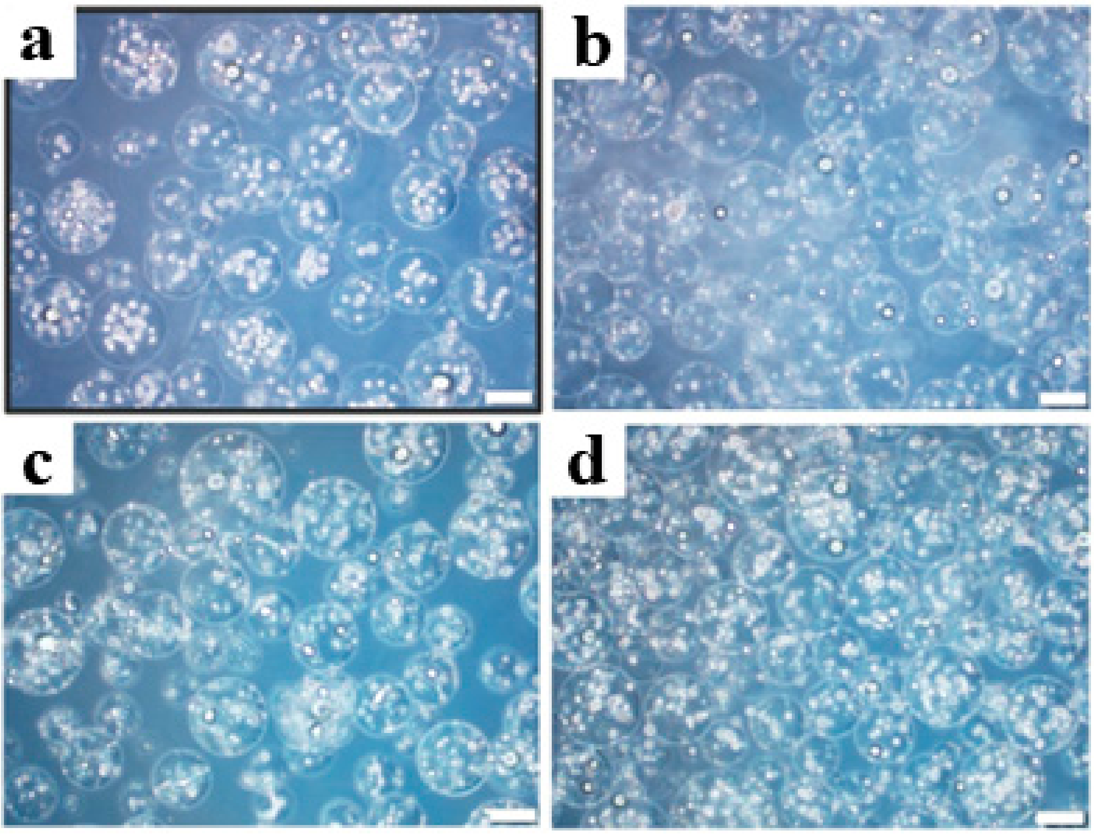

Figure S2. Photos of HSF and RAW246.7 cells encapsulated microspheres. a: HSF with density of 0.50 × 10^7/mL. b: RAW246.7 with density of 0.50 × 10^7/mL. c: RAW246.7 with density of 1.00 × 10^7/mL. d: RAW246.7 with density of 1.50 × 10^7/mL. Scale bar = 100 mm.

Table S1. Primer sequences for inflammatory regulation and reference genes.

Gene	Primer	Sequences (5′-3′)
Arg-1	Forward	TGTCCCTAATGACAGCTCCTT
	Reverse	GCATCCACCCAAATGACACAT
CD206	Forward	CTCTGTTCAGCTATTGGACGC
	Reverse	TGGCACTCCCAAACATAATTTGA
IL-10	Forward	TTTTCACAGGGGAGAAATCG
	Reverse	CCAAGCCTTATCGGAAATGA
bFGF	Forward	AGCGGCTGTACTGCAAAAA
	Reverse	TGCTTGAAGTTGTAATTTGATGTGT
HGF	Forward	GGAGGCAGCTATAAGGGAACA
	Reverse	AGCTCGAAGGCAAAAAGCTGTG
VEGF	Forward	CGAGGAGTTCAACGTCACCA
	Reverse	GCTCTGTCTTTCTTTGGTCTGC
IL-6	Forward	GCCGGCGGTGAATAATGAGA
	Reverse	TCGTCACTCCTGAACTTGGC
IL-8	Forward	CACTCCACACCTTTCCATCCC
	Reverse	CTTGCTGCTCAGCCCTCTTCA
TNF-a	Forward	TTGTCTACTCCCAGGTTCTCT
	Reverse	GAGGTTGACTTTCTCCTGGTATG
IL-1β	Forward	GGTGTGTGACGTTCCCATTA
	Reverse	ATTGAGGTGGAGAGCTTTCAG
CXCL9	Forward	AGGCACGATCCACTACAAATC
	Reverse	GCAGGTTTGATCTCCGTTCT
CCL5	Forward	GCCCACGTCAAGGAGTATTT
	Reverse	CTTGAACCCACTTCTTCTCTGG
CD86	Forward	TGCTCATCATTGTATGTCAC
	Reverse	GTCTCTCTGTCAGCGTTACT
CD206	Forward	CTCTGTTCAGCTATTGGACGC
	Reverse	TGGCACTCCCAAACATAATTTGA
iNOS	Forward	GGAATCTTGGAGCGAGTTGT
	Reverse	CCTCTTGTCTTTGACCCAGTAG
CXCL13	Forward	ATATGTGTGAATCCTCGTGCCA
	Reverse	GGGAGTTGAAGACAGACTTTTGC
β-actin	Forward	GGTGTGATGGTGGGAATGGG
	Reverse	ACGGTTGGCCTTAGGGTTCAG
GAPDH	Forward	AGGTCGGAGTGAACGGATTTG
	Reverse	GCCGTGGGTGGAATCATACT
